# Diffuse Idiopathic Pulmonary Neuroendocrine Cell Hyperplasia (DIPNECH) Progressing to Carcinoid Tumor: A Case of Chronic Cough

**DOI:** 10.7759/cureus.46659

**Published:** 2023-10-07

**Authors:** Olawale Babalola, Judge Muskrat, Venkatkiran Kanchustambham

**Affiliations:** 1 Pulmonology, University of North Dakota School of Medicine and Health Sciences, Fargo, USA; 2 Pulmonary Critical Care, University of North Dakota, Fargo, USA

**Keywords:** octreotide, tumorlets, chronic cough, lung wedge biopsy, carcinoid tumour, diffuse idiopathic neuroendocrine cell hyperplasia

## Abstract

Diffuse idiopathic pulmonary neuroendocrine cell hyperplasia (DIPNECH), a rare disease previously overlooked, is gradually being recognized as an important precursor state to pulmonary neuroendocrine tumors. The very insidious onset of symptom presentation makes early diagnosis of DIPNECH almost impossible in clinical settings. In this report, we present a case of persistent and worsening cough for over five years with waxing and waning lung nodules of varying sizes which were eventually diagnosed as DIPNECH on biopsy. However, due to the location and the multiplicity of these nodules, surgical resection was not an option in this case. The diagnostic workup including imaging and biopsy, management options, and possible prognosis of DIPNECH are discussed in detail. This report highlights the growing recognition of DIPNECH as a clinical entity to be aware of during the formulation of a differential diagnosis for patients presenting with chronic unrelenting cough and associated lung nodules.

## Introduction

Diffuse idiopathic pulmonary neuroendocrine cell hyperplasia (DIPNECH) is a rare disease that typically affects women in their fifth to sixth decade of life or even later. The clinical presentation of DIPNECH is variable and non-specific; however, persistent cough remains the most common presenting symptom. It is understood that the chronic cough is often the result of associated constrictive bronchiolitis and causes airflow limitations mimicking an obstructive lung disease pattern on spirometry [1]. As a result, some cases of DIPNECH might remain undiagnosed or misdiagnosed as asthma or chronic obstructive pulmonary disease (COPD). Therefore, a constellation of recurrent unrelenting cough, multiple small lung nodules on imaging, and mixed obstructive-restrictive lung disease pattern should prompt the clinician to include DIPNECH in differential diagnosis. The clinical course of disease is gradual with a minority of cases progressing to carcinoid tumors of the lungs [1]. While DIPNECH is now recognized as a precursor to pulmonary neuroendocrine tumors (NETs), the minimum criteria for diagnosis based on imaging is still yet to be ascertained, hence, biopsy remains the gold standard.

## Case presentation

**Figure 1:**
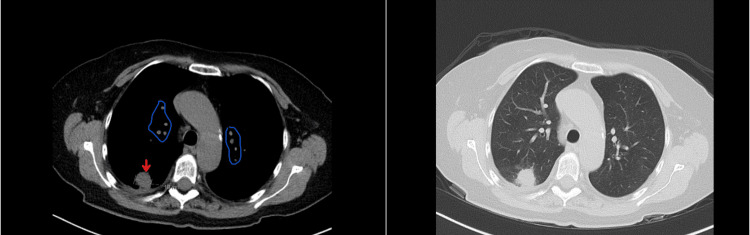
CT chest showing 18 mm nodule in right lower lobe (red arrow) and multiple smaller nodules bilaterally (encircled in blue) (left), high-density CT version of the same scan (right)

We present a case of a 74-year-old female with a history of Crohn's disease on Remicade infusion who was seen in the clinic for a follow-up of lung nodule and chronic cough. Historically, patients underwent chest CT for pulmonary symptoms 5 years prior which revealed multiple small (<9 mm) pulmonary nodules. These were surveilled with interval CTs, with no new changes until recently when a new irregular 19 mm nodule within the superior segment of the right lower lobe was seen (Figure 1). A PET scan was obtained which showed intrapulmonary nodules with relatively mild fludeoxyglucose F18 (FDG) activity, less than typically shown with a neoplastic process, and shrinkage of prior nodules. Given the reduction in size with a low pre-test probability of lung cancer, she was scheduled for a repeat CT in 3 months.

**Figure 2:**
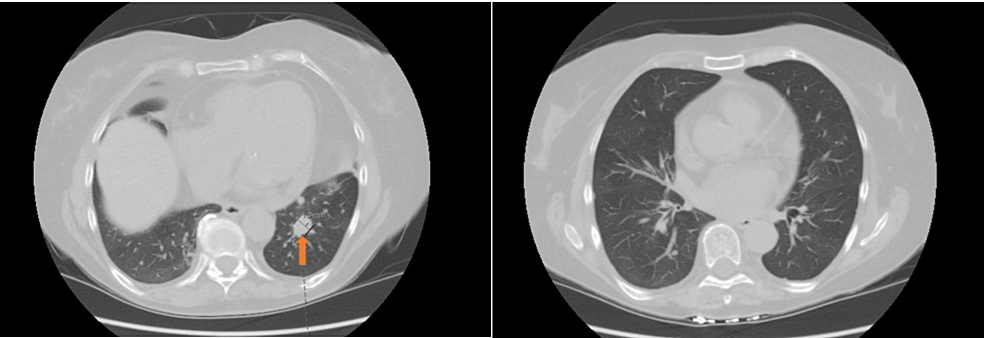
CT chest showing new 13 mm oblong mass in left lingula (left) and tree-in-bud pattern (right)

The repeat CT scan 3 months later showed complete resolution of the 18 mm right lower lobe lung nodule while prior smaller nodules remained stable. The patient was asymptomatic at this time. It was suspected that her lung nodules were likely post-infectious or inflammatory, especially in the setting of Crohn's disease. Yearly CT surveillance was initiated. Thirteen months later, chest CT revealed a new oblong left lower lobe lung mass just above the diaphragm (Figure 2). Given the on-and-off status of her lung nodules, the top differential considered was the Pulmonary manifestation of Crohn's disease-necrobiotic nodules. There were also worsening tree-in-bud opacities in the lingula thought to be due to secondary atypical infection given her immune status from Remicade infusion (Figure 2). She continued to have a cough at baseline, which was partly alleviated by Tessalon Perles, but no shortness of breath. 

Bronchoscopy was considered with a plan for bronchoalveolar lavage (BAL) to rule out infection. A pulmonary function test (PFT) was also planned to rule out obstructive lung disease. PFT was suboptimal due to severe obstructive ventilatory limitation with very narrowed airways thought to be due to her Crohn's disease, given her no smoking history. She was prescribed albuterol and fluticasone/vilanterol inhaler which improved her shortness of breath and almost alleviated her cough. Bronchoscopy showed no airway anomalies and BAL cultures for bacteria and fungi returned negative. Considering these results, a pulmonary manifestation of Crohn's disease-necrobiotic lung nodules remained the most suspected cause of her symptoms. Hence, she was started on a daily dose of 40 mg of prednisone for 4 weeks, and then 30 mg for 8 weeks followed by a tapering dose. Bactrim for pneumocystis pneumonia (PCP) prophylaxis was also initiated. She was scheduled for a repeat chest CT and clinic visit in 3 months.

During the 3-month follow-up visit, it was gathered that the patient stopped taking the prednisone a month short of the regimented period. She reported the return of severe cough and shortness of breath while on a tapered prednisone dose of 10 mg before abruptly stopping the medication. Repeat chest CT showed stable lung nodules. In the interim, she had been seen by primary care providers where azithromycin was prescribed for suspected community-acquired pneumonia. She reported feeling well while on the course of antibiotics but experienced an immediate return of respiratory symptoms once therapy antibiotics therapy was completed. The option to restart on 10 mg prednisone and reassess response was met with some degree of hesitation. The patient was more open to a trial of another 14-day course of azithromycin and transition to thrice weekly regimen for 6-12 months.

She initially had a good response but about 4 weeks into the regimen, her symptoms returned with worsening shortness of breath and persistent dry cough. We eventually adopted the plan to stop the azithromycin and restart the prednisone at 40 mg daily for 2 weeks followed by 20 mg for 10 weeks. Remicade for Crohn's disease was discontinued by Gastroenterology. Another follow-up was scheduled for 3 months. During this period, she had experienced significant tremor, weakness, changes in vision, and lightheadedness necessitating a reduction in prednisone dose. She also developed a non-occlusive deep vein thrombosis in the left lower extremity. A steroid-sparing agent, azathioprine, or mycophenolate mofetil, was considered to replace prednisone. CT chest, abdomen, and pelvis were obtained to rule out any malignancy, especially in the event of unprovoked deep venous thrombosis. CT scans returned stable for chest nodules and abdominopelvic scans were unremarkable.

**Figure 3:**
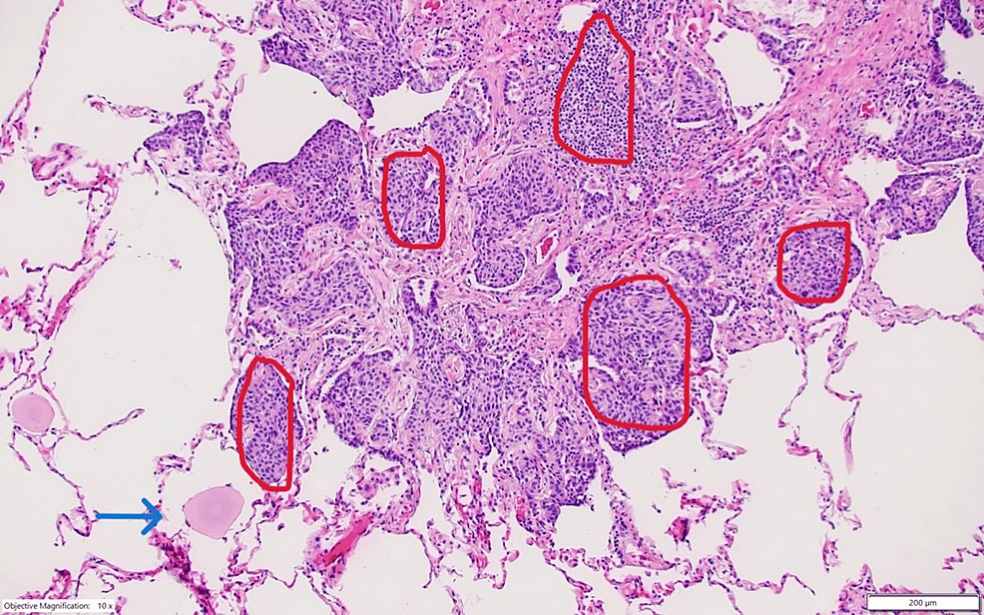
Diffuse proliferation of neuroendocrine cells (red circle) with areas of bronchiectasis (arrow) consistent with DIPNECH DIPNECH: diffuse idiopathic pulmonary neuroendocrine cell hyperplasia

**Figure 4:**
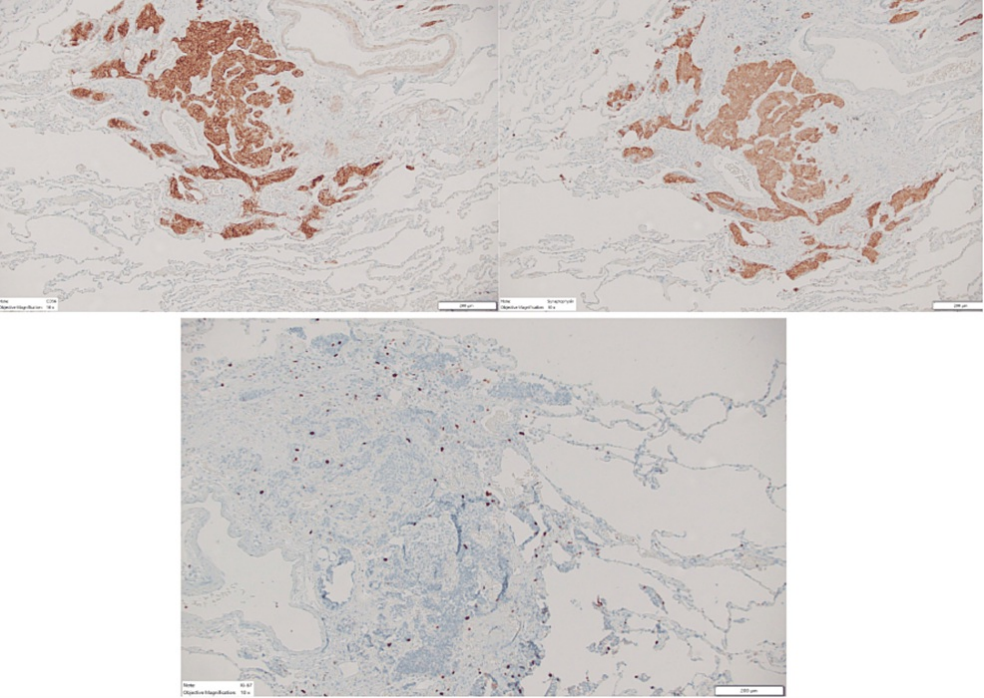
CD 56 (top left), synaptophysin (top right), and Ki-67 (lower) positive stains for neuroendocrine cells

Ultimately, a more invasive approach was considered to be appropriate in determining the cause of her chronic symptoms. Thoracoscopy with wedge biopsy of the pulmonary nodule was performed. The result showed multiple foci of neuroendocrine cell hyperplasia in small airways and carcinoid tumorlets, consistent with diffuse idiopathic pulmonary neuroendocrine cell hyperplasia (evaluated with submitted CK7, CD56, synaptophysin, chromogranin, p40, and Ki-67 stains) (Figure 3, 4). A referral was made to Oncology, and she was started on a monthly infusion of 20 mg of octreotide with great improvement in cough.

**Figure 5:**
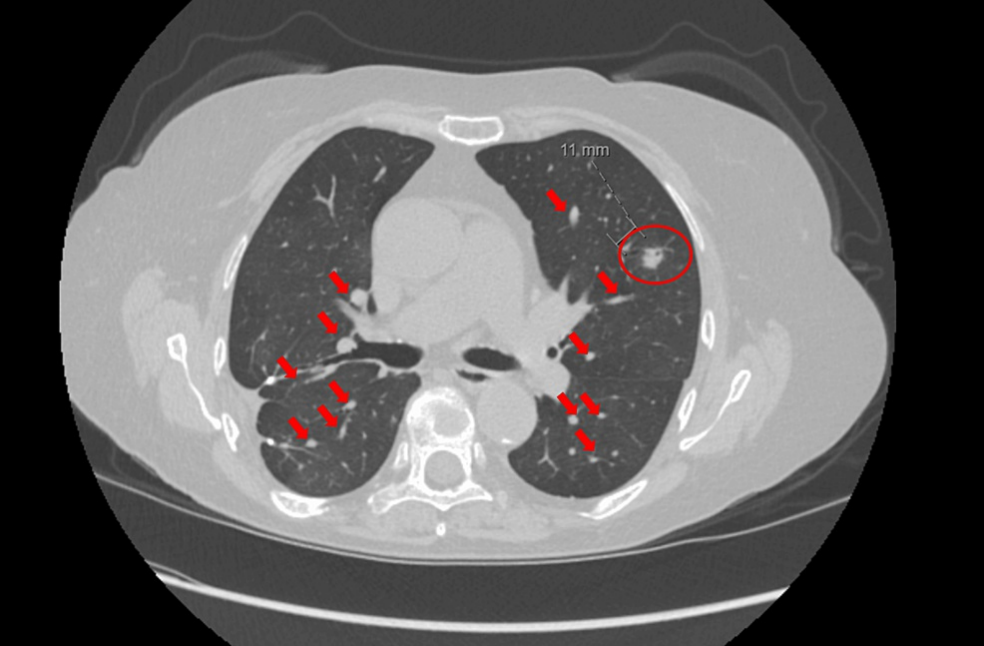
CT chest showing 11 mm left lung carcinoid tumor (circle) with multiple smaller carcinoid tumorlets (arrows)

Two years into octreotide therapy, her cough began to worsen. CT chest showed a new 11 mm left lung nodule (Figure 5). A PET scan was obtained for further evaluation which showed an 11 mm nodule in the central left lower lobe demonstrating increased FDG uptake likely representing low-grade malignancy/carcinoid. FDG PET/CT is of equivocal utilization in carcinoid neoplasm due to variable avidity, therefore, CT CT-guided biopsy was done which confirmed a carcinoid tumor. A dota-tate PET scan revealed multiple FDG avid nodules suspected to be active carcinoid tumors.

Tumors were determined to be unresectable. Due to multiple nodules and the location of the tumor, radiation therapy was not an option either. Everolimus was added to therapy given the progressive nature of the disease process. She continues to be followed by Oncology and Pulmonology with regular clinic visits and monthly octreotide for symptom control.

## Discussion

DIPNECH is a rare pulmonary disease that has been described by the WHO as “multifocal hyperplasia of neuroendocrine cells in bronchiolar epithelium, associated with multiple carcinoid tumorlets, with or without obliterative bronchiolitis and with or without a carcinoid tumor” [2]. DIPNECH is hypothesized by the WHO to be a pre-neoplastic lesion to pulmonary neuroendocrine tumors [3]. As it is such a rare disease, the precise incidence and prevalence is unknown. Histological studies have shown that DIPNECH may manifest as a proliferation of neuroendocrine cells either diffusely or linear, or as an aggregation known as neuroendocrine bodies [4]. Tumorlets and carcinoid tumors are defined as the proliferation of neuroendocrine cells beyond the basement membrane. Tumorlets are aggregations of neuroendocrine cells less than 5 mm in diameter while carcinoid tumors are nodules greater than 5 mm.

The pathophysiology of DIPNECH is poorly understood and is an area of potential study. There is no universal consensus on diagnostic criteria. Diagnosis is typically made years after symptom onset which can be misdiagnosed as asthma or followed by an incidental finding of a lung nodule on high-resolution chest CT then confirmed by histological review [5,6]. Symptoms can consist of progressively worsening cough, wheezing, and/or exertional dyspnea which is why it can be misdiagnosed as gastroesophageal reflux disease (GERD), COPD, or asthma. It has been proposed that five or more neuroendocrine cells in at least three separate small airways combined with three or more carcinoid tumorlets can be used to differentiate DIPNECH from other reactive airway diseases [7]. However, the applicability of this proposal to the clinical diagnosis of DIPNECH is yet to be ascertained.

Most patients diagnosed with DIPNECH are middle-aged women who are non-smokers. It is unknown whether genetics besides MEN1 are a factor or not, however, there is documentation of biological siblings who were both diagnosed with DIPNECH [8]. Lung function tests tend to reveal either obstructive, mixed obstructive/restrictive patterns, or normal patterns. There are some cases of rapidly progressive disease, but most patients experience a clinical course that is slowly progressive, as seen in our case.

Treatment and prognosis of DIPNECH depend on the severity of and progression of the disease, especially the extent of pulmonary fibrosis. Steroids, cytotoxic agents, somatostatin analogs, and surgical methods such as resection and transplantation have all been used for the treatment of DIPNECH. Data is lacking regarding efficacy and thus there is no universal standard for treatment. Treatment and prognosis will vary based on the individual and degree of neuroendocrine proliferation, such as evolution to carcinoid tumor which involves its own separate staging and treatment options [9].

## Conclusions

DIPNECH is a disease that has yet to have clearly defined risk factors, diagnostic criteria, treatment guidelines, or prognosis which leaves many avenues for future research to better understand this rare pulmonary disease. We have presented a case that supports the typical patient profile and clinical course previously described in other cases, which further delineates the need to include DIPNECH in the clinician’s differential diagnoses when a patient presents with chronic cough and lung nodules. DIPNECH has the potential to progress into unresectable carcinoid tumors, as highlighted in this case. Early detection may play an important factor in the treatment and prognosis of this insidious disease.
